# Book Reviews

**Published:** 1873-09-01

**Authors:** 


### Book Reviews.

Insanity in Its Relations to Crime. A Text and Commentary by Wm. A. Hammond, M.D. New York: D. Appleton & Co. For sale by W. B. Keen & Cooke, Chicago.

In this little volume the author quotes, first, three famous French murder cases, the details translated from the *causes celebres*, and examines and criticises them in his usual close, thorough manner, in order to determine whether these crimes were justly attributable to mental disease. The conclusion arrived at is, that although two of the accused were pronounced sane on the trial, and one of them hanged, they were, in Dr. Hammond's opinion, all three insane.

In his commentary, he touches upon one point which is too often overlooked in dealing with such cases. It is the power of self-control in the insane. This is by no means absent, as the public generally, and many physicians, seem to believe. This false impression is dangerous, for the insane are well aware of it, and they know that it gives them an immunity from the severest penalties of the worst crimes. Dr. Hammond urges insane criminals should be punished, not for the sake of society, directly, but for the sake of conveying such an impression to the other insane that they will exercise that power of self-control which they have, and which they can use if they try to do so.

Pharmaceutical Lexicon. A Dictionary of Pharmaceutical Science, containing a concise explanation of the various subjects and terms of Pharmacy. By H. V. Sweringen. Philadelphia: Lindsay & Blakiston. For sale by Jansen, McClurg & Co., Chicago. Price, cloth, $6.00; leather, $7.00.

This is a volume of 576 pages, and is issued in good style and binding. It is, we believe, the first attempt that has been made to form a Pharmaceutical Dictionary, and like most attempts in a new field, it falls considerably short of what the author designed to make it. In spite, however, of numerous evident deficiencies and imperfections, the work will, we feel confident, be highly prized, both by pharmacists and physicians, as affording a new and ready means of gaining information upon many of the innumerable and varied topics of professional inquiry.

An Introduction to the Study of Clinical Medicine, being a Guide to the Investigation of Disease, for the use of Students. By Octavius Sturges, M.D., etc. Philadelphia: H. C. Lea. Chicago: Jansen, McClurg & Co.

In this manual the author assumes no more than to point out to students *a method* —not the only, or necessarily the best method, he says, of interrogating patients at the bedside. He proceeds on the idea that "it is less by the use of subtle arts and intricate instruments, than in the exercise of common observation, *directed by method*, that the phenomena of disease are revealed."

It is a volume of only 127 pages, and is therefore of necessity exceedingly brief and condensed. It is, nevertheless, calculated, we believe, to be of much assistance to the student or young practitioner.

